# Parafoveal processing of repeated words during reading

**DOI:** 10.3758/s13423-021-02054-0

**Published:** 2022-02-25

**Authors:** Denis Drieghe, Robert Chan Seem

**Affiliations:** School of Psychology, University of Southampton, Southampton, SO17 1BJ UK

**Keywords:** Reading, Eye Movements and Reading, Parafoveal Processing, Repetition Effects

## Abstract

In an eye-tracking experiment during reading, we examined the repetition effect, whereby words that are repeated in the same paragraph receive shorter fixation durations. Target words that were either high-frequency or low-frequency words and of which the parafoveal preview was either correct or with all letters replaced were embedded three times in the same paragraph. Shorter fixation times and higher skipping rates were observed for high-frequency compared to low-frequency words, words for which the parafoveal preview was correct versus incorrect, and as the word was being repeated more often. An interaction between frequency and repetition indicated that the reduction in fixation times due to repetition was more pronounced for low-frequency words. We also observed influences of word repetition on parafoveal processing, as repeated words were skipped more often. An interaction between parafoveal preview and repetition indicated an absent repetition effect when the preview was incorrect, but this effect was short lived, as it was restricted to the first fixation duration on the target word.

When a word is presented more than once, processing time decreases. This *Repetition Effect* has been observed across several experimental paradigms such as lexical decision (e.g., Scarborough et al., 1977), naming (e.g., Masson & Freedman, 1990), and tachistoscopic word identification (Humphreys et al., 1988). Indeed, studying word repetition has been important not just for psycholinguistic but also memory research (e.g., Scarborough et al., 1977). Raney and Rayner (1995) examined repetition effects during reading by tracking eye movements of participants who read a passage and then immediately reread the same passage. They observed an overall speedup in the second reading which was reported before (e.g., Hyönä & Niemi, 1990). Raney and Rayner additionally compared the reading times of embedded high-frequency and low-frequency words. High-frequency words typically receive shorter fixation times than low-frequency words (Rayner & Duffy, 1986)—a robust finding called the *Frequency Effect*. Interestingly, whereas single word recognition studies (e.g., lexical decision) observed that low-frequency words show larger repetition effects than high-frequency words (e.g., Scarborough et al., 1977), this interaction between frequency and repetition was not observed by Raney and Rayner, who observed main effects only of frequency and repetition. Chamberland et al. (2013) also observed additive effects when the same text was read more than once. However, in a subsequent analysis of the Raney and Rayner data, Rayner et al. (1995) examined repetition effects of words within the initial reading of the passages, and although they did not present formal statistics, the reported means strongly indicated an interaction between frequency and repetition. This interaction, observed when only the words and not the entire text was reread, showed that fixation times on low-frequency words were indeed reduced more compared to high-frequency words when repeated. Reading times were close to identical for high-frequency and low-frequency words upon the third reading. We can only speculate why repetition effects are different when the same text is reread, but substantial changes in how predictable words in the text will be upon rereading identical text are likely. The current paper will reexamine frequency and repetition effects of words repeated within the same passage and will additionally examine the time course of these effects.

Since Rayner et al. (1995), other studies have also observed this interaction between frequency and repetition. Lowder et al. (2013) examined reading times of proper names that were repeated in a sentence. Analyses of the names of incoming freshmen were used to identify high-frequency (e.g., *Stephen*) and low-frequency (e.g., *Dominic*) proper names. Besides main effects of frequency and repetition such that high-frequency and repeated words were read comparatively faster, they also observed an interaction in that repetition effects were larger for the low-frequency proper names. This was true both for first-pass fixation times and skipping rates. Kamienkowski et al. (2018) studied eye movements on repetitions of words within natural text (i.e., not experimentally manipulated). In their corpus study, they also observed the interaction between frequency and repetition such that fixation durations in low-frequency words decreased upon being repeated, but no repetition effects were observed in high-frequency words.

Theoretical interpretations of the repetition effect within single word recognition vary according to different models. Within classic logogen models (Morton, 1969), for instance, repetition effects are thought to originate from a change of activation or threshold due to a recent occurrence of the repeated word. Whereas a detailed discussion of these models is outside the scope of this brief report, two comments need to be made. First, most models in one form or another assume that the interaction between frequency and repetition is due to high-frequency words being encountered so often that a single additional encounter will not influence the speed of retrieval much, whereas there is opportunity for a substantial speedup for low-frequency words. Second, when thinking about repetition effects of a word embedded in a paragraph compared with single word experiments, any theoretical interpretation will need to consider that the repetition of a word will likely influence its predictability. Predictability is typically assessed by presenting participants, who do not partake in the eye-tracking experiment, the sentences up to but not including the target word. They are then asked to continue the sentence. A word often used as a continuation is considered to be predictable from the preceding context. Effects of predictability are well established on eye movements during reading such that highly predictable words are skipped more often and are fixated for less time compared to words that are not predictable from the preceding context (Balota et al., 1985). It is reasonable to assume that at least part of the repetition effect in text reading is due to changes in predictability as with multiple presentations participants might anticipate a reoccurrence of the target word.

During reading, readers pick up information not just from the currently fixated word but also from the next word in the sentence, a word that will typically be located in the parafoveal area of the visual field. This parafoveal processing is most clearly demonstrated by means of the gaze-contingent boundary paradigm (Rayner, 1975). In this paradigm an invisible boundary is located before the target word. Once the eyes cross the invisible boundary a preview is replaced by the actual target word. Because the display change happens during a saccade when the eyes are functionally blind, participants are typically unaware of the display change switching the preview to the target word. The *Parafoveal Preview Benefit* is the observation that fixation times on the target word are shorter when the preview was identical to the target word compared with when letters were replaced. This paradigm has resulted in many insights into the nature of information that is being extracted in the parafovea (see Schotter et al., 2012, for a review) and also allows for the observation of some of the earliest influences of word processing in the eye-tracking record (i.e., information extracted even before the eyes land on it), which is why this technique provides a window on the earliest stages of word recognition. In the current paper, we used the boundary technique to determine the earliest influence of word repetition in the eye-tracking record.

The present study had participants read paragraphs that have embedded target words that were manipulated in three ways: The target words were either high frequency or low frequency, the preview before the eyes landed on them was either identical to the target word or with all letters replaced, and the words were embedded three times in the paragraph, allowing observations for the first, second, or third presentation. This experiment allowed us to examine two phenomena concerning repetition effects during reading:

- The interaction between frequency and repetition such that fixation times on low-frequency words are reduced to a higher extent when the word is repeated compared to high-frequency words. Even though this interaction has been observed previously, it merits to be established within experimentally controlled sentences (as opposed to natural text within a corpus study), with content words as target words and with proper statistical analysis. This is especially important given observations of both additive and interactive effects in single words studies (e.g., Kinoshita, 2006), although there is no strong reason to assume why we would not replicate the interactive relationship between frequency and repetition reported by Rayner et al. (1995).
- The time course of the repetition effect. Is there an interaction such that the processing of the parafoveal preview is influenced by whether the target word will be presented for the first, second, or third time? Any such effect would indicate repetition effects influencing very early (i.e., parafoveal) word processing during reading. If an interaction exists, we would predict the direction to be such that repetition effects would lead to faster processing of the target word especially when processing of the preview is allowed in the parafovea, analogous to findings of a larger preview benefit for predictable compared to unpredictable target words (e.g., Balota et al., 1985).

## Method

### Participants

Forty-five native English speakers with normal or corrected-to-normal vision and no known reading difficulties were recruited from the University of Southampton in return for course credit. Participants were asked after the experiment whether they noticed anything strange whilst reading. None mentioned the repetition of words. Two participants were excluded for noticing too many display changes (10 or more), one participant because we did not record whether the participant noticed display changes and one participant for proudly proclaiming they were skim reading after the experiment. In total, the data of 41 participants were analyzed. The number of participants was selected to exceed the numbers of participants used in previous studies looking into the interaction between the frequency and repetition effect of a word being repeated in the same paragraph (see Introduction).

### Apparatus

An SR Research EyeLink 1000 system was used to capture eye movements. Whilst viewing was binocular, only movements from the right eye were recorded. Paragraphs consisted of several single line sentences presented as either five or six lines of text. All stimuli were displayed on a 21-in. Viewsonic CRT monitor with a display resolution of 1,024 × 768 pixels and a refresh rate of 100 Hz. All text was presented in black monospaced Courier New 13-pt font on a light-grey background and was in lowercase, except where capitals were appropriate. The participant’s eyes were approximately 65 cm from the monitor. At this distance, three characters equaled about 1° of visual angle.

### Stimuli and design

The stimuli consisted of 32 paragraphs of which 16 were five lined, and 16 six lined. Each line of text was constructed as a single sentence of 85 to 90 characters in length, with a vertical separation of 96 pixels between them. Target words were presented three times per paragraph, positioned to either the middle-left, middle, or middle-right of the sentences, and they featured on lines 1, 3, and 5 for five-line paragraph frames, or lines 2, 4, and 6 for six-line paragraph frames. Word frequencies of the target words were established. Target words were all five-letter long nouns and were either high-frequency (average 4.96 Zipfian Log frequency) or low-frequency words (average 2.99 Zipfian Log Frequency; Van Heuven et al., 2014). A *t* test indicated the difference in frequency was significant: *t*(31) = 18.62, *p* < .001. Using the gaze-contingent boundary paradigm, target word previews were presented either normally, or incorrectly. Incorrect target previews were created by replacing each letter to form a nonpronounceable nonword.^1^ To preserve visual similarity of the preview to the target, ascenders, neutrals, and descenders were each replaced with their corresponding letter type. Once the eyes crossed the invisible boundary preceding the space before the target word, the display changed to reveal the correct target orthography. An example of the stimuli is shown in Table 1. All the materials are available online (https://osf.io/gytrz/).

**Table 1:** Example of the stimuli

It was an incredible atmosphere, raucous noise filled the air with a sense of excitement.	
This was my first ##### since moving to the South, people knew how to put on a show here.	
Looking around the crowd, people exuded this amazing carefree vibe, it was refreshing.	
I’d mostly been ambivalent about the idea of a loud ##### but I was having a great time.	
Having spent my life in the countryside, my idea of fun was a coffee with a good book.	
Something about this place brought out the best in me, this ##### made me feel alive.	

Note. The ##### indicates the location of the targets for which the previews were *party* (High-frequency–Correct preview), *ynsfp* (High-frequency–Incorrect preview), *rodeo* (Low-frequency–Correct preview), and *vchwx* (Low-frequency–Incorrect preview)

The sentence context preceding the first encounter of the target word was constructed to be neutral, that is, nonpredictive. This was determined through the use of a cloze task where participants read a partial sentence and were asked to report which word they believed followed (e.g. “The quarrelsome student responded with his usual . . .”). The cloze task was completed by 10 participants who did not partake in the eye-tracking experiment. For 29 pairs, neither the low-frequency nor the high-frequency word was ever used as a completion for the sentence. Removing the remaining three pairs (one pair in which the high-frequency word was used once as a completion, one pair in which the high-frequency word was used twice, and one pair in which the high-frequency word was used four times and the low-frequency word once) did not change any of the data patterns, so we decided to keep them in the analyses. Note that a highly predictable word would typically get a sentence completion ratio of >70% (Rayner & Well, 1996), which is considerably higher than the ratio we observed for any word pair.

The design was a 2 × 2 within-subjects design, where target word frequency was manipulated on two levels (high frequency and low frequency), target preview on two levels (normal and incorrect preview), and number of target presentations (i.e., the first, second, or third time) was treated as a continuous variable. Four separate counterbalanced lists were created, each containing 32 experimental paragraph-frames of the varying four conditions. Thus, while each participant read 32 experimental paragraph frames, they saw only one of the four possible versions of each paragraph. An additional 16 filler paragraph frames (8 five line, and 8 six line) were mixed in with the 32 experimental paragraphs, and all were presented in a pseudorandom order preceded by five practice paragraphs.

### Procedure

Participants were told that they would be reading paragraphs of text off of a monitor, which they would be reading for comprehension. They were informed these paragraphs would often be followed by a comprehension question, and to respond to these yes/no questions using the provided button box. Prior to the study, the participant’s head was stabilized using a chin and forehead rest. Participants were then put through a calibration and validation procedure. This process entailed a nine-point grid of dots that appeared individually on-screen, where the participant’s role was to fixate these dots in turn as they appeared. The study allowed for a maximum average error of 0.5° of visual angle. In our lab, studies using boundary paradigms typically allow for a maximum error of 0.3° of visual angle (or one character) during calibration. However, it is worth noting our previous experiments have been conducted using centrally positioned single sentences. For multiline experiments, this criterion is not workable, as error is present on two dimensions (width and height) as opposed to one. Preceding the display of paragraph frames, participants fixated a centrally located drift correct, then a second one located to the top-left of the screen. The second drift correct was positioned in the same location as the first letter of the first word of the paragraph frames, so as the drift correct was finished, the participant was fixating the first word in the paragraph. Once the participant had finished reading the paragraph, they pressed a button on a response pad. Comprehension questions followed 37 out of 57 trials (70%), and were simple “yes” or “no” questions, for which the participant responded with the left-hand button for yes, and the right-hand button for no. The accuracy in answering the comprehension questions was 92.55%. In total, the experiment lasted approximately 45 minutes. Participants were allowed to take breaks throughout the experiment.

### Results

Following standard procedures using the Data Viewer software’s clean function (SR Research Ltd., Ontario, Canada) all fixations below 80 ms and within 0.33° (one character) of another fixation below 80 ms were combined; any fixation below 40 ms was combined with any others below 40 ms within 1.25°; and finally, any remaining fixations shorter than 80 ms or longer than 800 ms were removed. Further trials were removed if a display change was incurred for any of the following three reasons: (1) a preboundary fixation incurring a display change; (2) the display change was triggered by a “hooking” saccade, whereby the eyes cross the boundary before hooking back and landing before the boundary; (3) display changes that were triggered late into a fixation (more than 10 ms in). Each continuous dependent measure per participant was additionally checked for any remaining outliers—any observations more than three standard deviations from the grand mean were removed. In total, 26% of trials were excluded. Data loss was similar across conditions. Data loss was higher than is typically observed in single sentence boundary change experiments presumably because it was not feasible to be as strict as usual on the accuracy of the calibration. We will return to this issue in the Discussion.

As we were interested in parafoveal processing, we focused on measures during first pass (i.e., before the eyes move past the target word). The dependent measures used were *first fixation duration* (FFD; the duration of the initial first-pass fixation on the word); *single fixation duration* (SFD; the duration of a fixation given that exactly one fixation is made); *gaze duration* (GD; the sum of all first-pass fixations); *go-past time* (the sum of all fixations made before making a saccade to the right of the target word, including regressions to earlier sections of text); and lastly, *skipping probability* (the probability that the target word is not fixated during first-pass reading). Fixation durations were log-transformed to increase the normality of their distributions.

The data were analyzed using linear mixed-effects models (LMM) with the lme4 package (Version 1.1-27.1; Bates et al., 2015) in R (Version 4.1.0; R Core Team, 2021). Contrasts for the effects of frequency and preview were specified as −.5/.5. Presentation was centred and entered as a continuous variable. A full random structure (see Barr et al., 2013) was specified for the random factors subjects and stimuli. These models were trimmed until a reliable convergence was achieved in the absence of perfect correlations in the random structure. Trimming happened by removing interactions or slopes, starting with the one associated with the smallest variance, and so on. As high degrees of freedom are typical for LMMs, the *t* statistic approximates the *z* statistic—thus, absolute values equal to or greater than 1.96 were considered significant. Skipping analyses were specified the same as the aforementioned LMMs, but with the use of logistic GLMMs. Descriptive means and standard deviations for each condition per presentation are presented in Table 2, and the LMM and GLMM results are presented in Table 3.

**Table 2:** Means (and standard deviations) of eye-movement measures calculated across subjects for target words as a function of presentation, preview type, and word frequency

Presentation	Measure	Normal preview		Incorrect preview	
		HF	LF	HF	LF
First	FFD (ms)	226.77 (43.26)	248.45 (42.30)	238.57 (42.08)	261.47 (40.66)
	SFD (ms)	229.16 (52.50)	265.56 (59.94)	251.61 (51.95)	286.55 (53.43)
	GD (ms)	251.21 (52.37)	289.56 (67.46)	271.83 (55.30)	314.51 (68.50)
	Go-past time (ms)	268.97 (53.78)	317.57 (77.76)	309.32 (79.31)	356.07 (79.50)
	Skipping (%)	19.93 (19.29)	13.04 (12.00)	14.02 (13.46)	10.25 (10.68)
Second	FFD (ms)	215.59 (44.96)	230.88 (53.34)	248.50 (58.21)	245.61 (53.39)
	SFD (ms)	217.23 (47.50)	240.32 (64.15)	257.43 (62.76)	274.62 (88.12)
	GD (ms)	223.91 (49.65)	258.66 (74.09)	274.89 (69.73)	279.88 (80.11)
	Go-past time (ms)	248.19 (55.49)	273.92 (82.90)	287.60 (78.22)	306.28 (82.68)
	Skipping (%)	23.39 (23.43)	18.72 (23.18)	20.32 (20.92)	15.89 (18.95)
Third	FFD (ms)	214.69 (42.70)	222.46 (36.98)	241.06 (41.35)	248.28 (55.64)
	SFD (ms)	212.77 (46.09)	225.01 (53.46)	243.39 (48.53)	260.65 (70.60)
	GD (ms)	223.39 (41.96)	241.48 (49.05)	254.06 (42.59)	269.99 (62.24)
	Go-past time (ms)	235.03 (48.87)	261.43 (55.70)	285.63 (79.04)	295.09 (68.81)
	Skipping (%)	28.70 (25.70)	27.26 (24.22)	27.47 (24.11)	20.24 (21.57)

**Table 3:** Linear mixed models of the eye-movement measures on the target words

Measure	Fixed effect	β	*SE*	*t*/*z*
FFD	Intercept	5.41	0.02	**302.92**
	Preview	−0.08	0.02	**−5.32**
	Frequency	0.06	0.02	**2.83**
	Presentation	−0.02	0.01	**−2.12**
	Preview × Frequency	0.04	0.03	1.51
	Preview × Presentation	−0.03	0.02	**−1.98**
	Frequency × Presentation	−0.04	0.02	**−2.43**
	Preview × Frequency × Presentation	0.03	0.03	0.81
SFD	Intercept	5.44	0.02	**243.01**
	Preview	−0.12	0.02	**−6.01**
	Frequency	0.09	0.02	**3.69**
	Presentation	−0.04	0.01	**−3.39**
	Preview × Frequency	0.03	0.03	1.08
	Preview × Presentation	−0.03	0.02	−1.76
	Frequency × Presentation	−0.04	0.02	**−2.44**
	Preview × Frequency × Presentation	0.00	0.04	0.08
GD	Intercept	5.50	0.02	**254.86**
	Preview	−0.12	0.02	**−6.25**
	Frequency	0.10	0.02	**4.01**
	Presentation	−0.06	0.01	**−4.73**
	Preview × Frequency	0.06	0.03	**1.99**
	Preview × Presentation	−0.02	0.02	−1.10
	Frequency × Presentation	−0.04	0.02	**−2.24**
	Preview × Frequency × Presentation	0.03	0.04	0.95
Go-past time	Intercept	5.58	0.02	**237.94**
	Preview	−0.13	0.02	**−6.07**
	Frequency	0.11	0.02	**4.83**
	Presentation	−0.07	0.01	**−7.39**
	Preview × Frequency	0.05	0.03	1.60
	Preview × Presentation	−0.01	0.02	−0.32
	Frequency × Presentation	−0.04	0.02	**−2.10**
	Preview × Frequency × Presentation	0.02	0.04	0.45
Skipping proportion	Intercept	−1.67	0.15	**−11.21**
	Preview	0.27	0.10	**2.64**
	Frequency	−0.33	0.10	**−3.25**
	Presentation	0.43	0.06	**7.05**
	Preview × Frequency	−0.03	0.20	−0.15
	Preview × Presentation	−0.04	0.12	−0.36
	Frequency × Presentation	0.09	0.12	0.79
	Preview × Frequency × Presentation	0.24	0.24	1.02

|*t*| or |*z*| ≥ 1.96 are considered significant and are reported in bold typeface. The random structure for FFD was (1 + preview + frequency|subject) + (1 + frequency + presentation|item), for SFD it was (1 + preview + frequency|subject) + (1 + preview + frequency + presentation|item), for GD it was (1 + preview + frequency|subject) + (1 + frequency + presentation|item), for go-past times it was (1 + preview|subject) + (1 + frequency|item), and for word skipping it was (1|subject) + (1 |item)

#### Fixation times

**Fig. 1:**
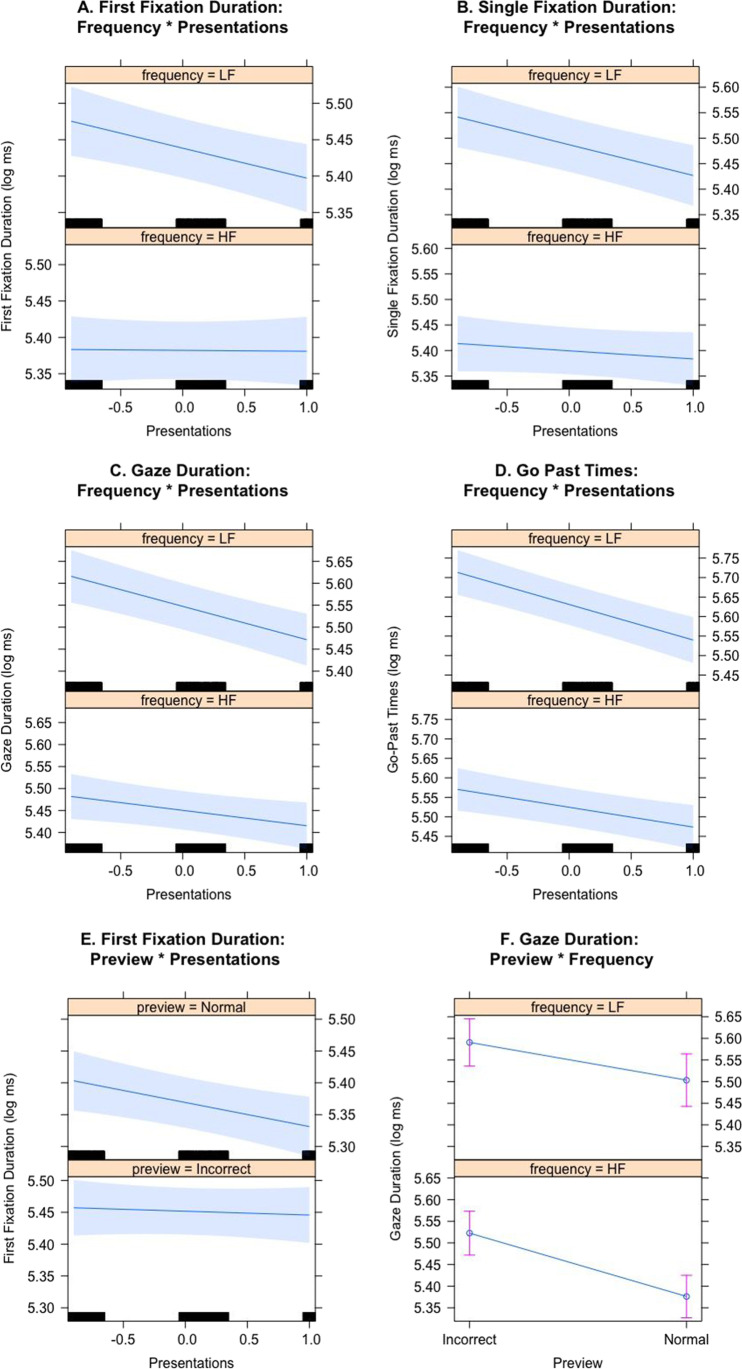
The Frequency × Number of Presentations interaction in first fixation duration (**a**), single fixation duration (**b**), gaze duration (**c**), and go-past times (**d**). The Preview × Number of Presentations interaction for first fixation duration (**e**). The Preview × Frequency in Gaze Duration interaction (**f**). The grey bands represent 95% confidence intervals except in (**f**), where error bars are shown. Note the number of presentations was centered (so −1 is first presentation, 0 second presentation, and +1 third presentation)

In all fixation time measurements, the three main effects were significant such that fixation durations were shorter when the preview was correct compared to when it was incorrect, and when the target was high frequency compared to low frequency. Fixation durations also became shorter as the target word was being repeated. An interaction between frequency and presentations was observed for all fixation time measures such that the effect of reduced fixation durations upon repetition was more pronounced for low-frequency words (see Fig. 1a–d). Two additional interactions were observed. In first fixation duration, an interaction was observed between preview and presentations (see Fig. 1e), such that preview benefit (i.e., the difference between normal and incorrect preview) became bigger as the number of presentations went up. This was due to number of presentations reducing first fixation time but not when the preview was incorrect. Finally, in gaze duration an interaction between preview and frequency (see Fig. 1f) indicated a bigger frequency effect when the preview was normal.

#### Skipping probability

We observed only main effects in skipping probability such that high-frequency target words were skipped more often than low-frequency words, and that correct previews triggered more skipping of the target words than incorrect previews. As the target word was repeated more often, it was also skipped more often.

## Discussion

In this reading experiment, we set out to examine two phenomena. Firstly, the interaction between the frequency and the repetition effect, and secondly, the time course of the repetition effect. Our results were straightforward. First of all, our three main effects were observed in all fixation times measures and in skipping rates such that fixation times were shorter and skipping rates were higher for high-frequency compared to low-frequency words, when the preview was identical to the target word versus when the preview was altered and as the target word was repeated more often.

We also observed the predicted interaction between frequency and presentation such that the reduction in fixation times due to repetition is more pronounced for low-frequency words. This interaction replicates earlier findings (Kamienkowski et al., 2018; Lowder et al., 2013; Rayner et al., 1995) but on nouns embedded in experimentally controlled sentences and properly analyzed. This interaction is typically interpreted as the processing speed of high-frequency words being relatively immune to repetitions as they are already encountered often whereas low-frequency words allow for considerable speedup. Indeed, the Kamienkowski et al. (2018) data indicate no repetition effects whatsoever in gaze durations on high-frequency words, whereas Rayner et al. (1995) indicated some reduction in gaze duration. Our results (compare Fig. 1a to 1d) suggest a pattern in which repetition effects on high-frequency words gradually appear in the eye-tracking record with no clear effect in first fixation duration, some effect starting to appear in single fixation duration, getting stronger in gaze duration, and becoming quite pronounced in go-past times.

Our second research focus was on a potential interaction between the repetition effect and parafoveal preview benefit. Such an interaction would indicate a very early influence of repetition on word processing. We observe such an interaction in that repetition reduced fixation times, but only when the preview was correct and not when the preview was incorrect. Note that this observation is similar to findings by Balota et al. (1985) who observed a predictability pattern in gaze duration that was absent when the preview was visually dissimilar. However, in our experiment this effect was short lived, as it only appeared in first fixation duration and was not close to significant in any of the other measures. It would appear a very limited early effect exists in that the processing of the incorrect preview briefly prevents repetition benefits. Importantly, a more pronounced influence of repetition on parafoveal processing was observed on word skipping. Word skipping is based on parafoveal processing and the repetition of a target word did lead to an increase in word skipping, showing an early influence of the repetition effect on parafoveal processing in terms of saccade target selection.^2^

Additionally, an interaction was observed in gaze duration between preview and frequency such that bigger frequency effects were observed when the preview was normal. This interaction was also reported by Degno et al. (2019) but was in their experiment restricted to first and single fixation duration. Clearly, the correct preview allows lexical processing to begin, but not for the incorrect preview, although in our data the effect is less pronounced as it just reached significance (*t* = 1.99) and only in gaze duration. This less pronounced and maybe also later appearance of the interaction could be due to our experiment examining the reading of paragraphs, whereas Degno et al. examined the reading of single sentences. Lexical influences have been shown to be less pronounced in eye movements in paragraph reading compared with single sentences (e.g., frequency effects; Radach et al., 2008).

As we indicated in the Introduction, the repetition effect in all likelihood will be at least partially explained by a higher predictability of the repeated word. Whereas there is no reason to assume this would not be the case in the current experiment as well, there are some indications that this overlap will not be 100%, as the interaction between frequency and repetition on the one hand and the interaction between frequency and predictability on the other hand manifest themselves quite differently. Whereas the former is clearly interactive in our data, the data on the latter suggests an additive relation between frequency and predictability (Rayner et al., 2004; although see Hand et al., 2010, for a suggestion that this might depend on launch site). Moreover, whereas repetition effects for high-frequency words are absent or limited, clear predictability effects still occur for high-frequency words (Rayner et al., 2004). Even though our experiment is clearly not set up to tease apart effects of repetition and predictability, we hope to see experiments explore this interesting relation in the future.

Few studies so far have implemented the eye-contingent boundary technique during paragraph reading as opposed to single sentences. Kaakinen and Hyönä (2014) reported 24% overall data loss (with slightly different exclusion criteria), whereas we lost 26%, a high number we attribute to difficulties maintaining the usually more stringent criteria employed in single sentence studies on average error in the calibration. We speculate that our data loss might be even slightly higher than Kaakinen and Hyönä because we had boundaries triggering display changes closer to the edges of the screen whereas they only had one boundary per page placed relatively close to the middle of the screen.^3^ We therefore caution fellow researchers concerning putting boundaries for display changes too far away from the middle of the screen.^4^

Summarizing, we observed the anticipated interaction between frequency and repetition such that repetition effects were more pronounced for low-frequency words. Repeated words were also skipped more often, indicating that repetition influences parafoveal processing, although an increased preview benefit effect due to repetition was short-lived and only observed in first fixation duration.

## Footnotes

1 Orthographic repetition blindness is the phenomenon observed in visual word recognition by which a second occurrence of a repeated word or a word visually similar to the repeated word is not reported (e.g. Harris & Morris, 2000). This would not apply to our unrelated condition, as all letters were replaced so the unrelated condition was not visually similar.

2 Another potential early effect in the eye-tracking record are so-called parafoveal-on-foveal effects, whereby the processing of word *n* + 1 influences the fixation times on word *n*. The existence of lexical parafoveal effects is contentious especially in the context of controlled experiments (Brothers et al., 2017; for a review, see Drieghe, 2011). No significant effects were observed on the word preceding the target word in the current experiment (all *t*s < 1.68).

3 Personal communication, J. K. Kaakinen, March 9, 2021.

4 Note that even though data loss was substantial, post hoc power analyses using the simr package (Green & MacLeod, 2016) still determined the power for our three main effects in gaze duration to be well above .90.
